# P-934. Emerging Trends and Challenges in Pediatric Infective Endocarditis: Insights from a Single-Center Observational Study

**DOI:** 10.1093/ofid/ofae631.1125

**Published:** 2025-01-29

**Authors:** Santhanam Naguthevar, Vibhor Tak, Daisy Khera, Siyaram Didel, Kumar S Abhishek

**Affiliations:** AIIMS JODHPUR, Jodhpur, Rajasthan, India; AIIMS Jodhpur, Jodhpur, Rajasthan, India; AIIMS, Jodhpur, Rajasthan, India; AIIMS, Jodhpur, Rajasthan, India; AIIMS, Jodhpur, Rajasthan, India

## Abstract

**Background:**

Infective endocarditis (IE) in children is rare but challenging, with an incidence of 0.43–0.69 cases per 100,000 children–year. Risk factors include congenital and acquired heart diseases, as well as healthcare-associated acquisition via prosthetic devices

**Methods:**

A prospective study was conducted at AIIMS Jodhpur's Department of Medicine from January 2021 to 2024. It included pediatric patients with suspected infective endocarditis (IE).

**Results:**

In this study, the most common age for pediatric endocarditis was 16-20 years, representing 50% of the patients. Following that, the 11-15 years age group accounted for 20% of cases. Both the 0-5 years and 6-10 years age groups each constituted 15% of the patients.

In the study, 65% of pediatric endocarditis cases were male, and 35% were female. Additionally, 60% of patients had a previous heart disease, while 35% did not. For endocarditis type, 15% were prosthetic IE, and 85% were native valve cases.

In the study, the mitral valve was most commonly affected, accounting for 65% of pediatric endocarditis cases. The aortic valve was involved in 20% of cases, while the tricuspid valve was affected in 10%. A combination of aortic and pulmonary involvement was observed in 5% of the cases.

In the study, culture-negative cases were the most common, accounting for 40% of pediatric endocarditis cases. Following that, both Streptococcus and Burkholderia each represented 15% of the infections. Staph aureus and Libmansack IE were each responsible for 10%, while Acinetobacter baumannii and Bartonella comprised 5% of the cases.

In the study, 80% of pediatric endocarditis patients showed improvement, while 20% unfortunately expired.

**Conclusion:**

This study emphasizes the diagnostic challenges of pediatric IE, with many cases culture-negative and caused by diverse organisms. Predominantly affecting adolescents and those with pre-existing heart conditions, IE in children presents multifaceted complexities, underscoring the need for improved diagnostic and treatment strategies to achieve successful outcomes.

**Disclosures:**

**All Authors**: No reported disclosures

